# Induction of osteoblast apoptosis stimulates macrophage efferocytosis and paradoxical bone formation

**DOI:** 10.1038/s41413-024-00341-9

**Published:** 2024-08-05

**Authors:** Lena Batoon, Amy Jean Koh, Susan Marie Millard, Jobanpreet Grewal, Fang Ming Choo, Rahasudha Kannan, Aysia Kinnaird, Megan Avey, Tatyana Teslya, Allison Robyn Pettit, Laurie K. McCauley, Hernan Roca

**Affiliations:** 1 Department of Periodontics and Oral Medicine, University of Michigan, School of Dentistry, Ann Arbor, MI 48109 USA; 2 Mater Research Institute, The University of Queensland, Brisbane, QLD 4102 Australia; 3 Department of Pathology, University of Michigan, Medical School, Ann Arbor, MI 48109 USA

**Keywords:** Bone, Bone quality and biomechanics

## Abstract

Apoptosis is crucial for tissue homeostasis and organ development. In bone, apoptosis is recognized to be a main fate of osteoblasts, yet the relevance of this process remains underexplored. Using our murine model with inducible Caspase 9, the enzyme that initiates intrinsic apoptosis, we triggered apoptosis in a proportion of mature osteocalcin (OCN^+^) osteoblasts and investigated the impact on postnatal bone development. Osteoblast apoptosis stimulated efferocytosis by osteal macrophages. A five-week stimulation of OCN^+^ osteoblast apoptosis in 3-week-old male and female mice significantly enhanced vertebral bone formation while increasing osteoblast precursors. A similar treatment regimen to stimulate osterix^+^ cell apoptosis had no impact on bone volume or density. The vertebral bone accrual following stimulation of OCN^+^ osteoblast apoptosis did not translate in improved mechanical strength due to disruption of the lacunocanalicular network. The observed bone phenotype was not influenced by changes in osteoclasts but was associated with stimulation of macrophage efferocytosis and vasculature formation. Phenotyping of efferocytic macrophages revealed a unique transcriptomic signature and expression of factors including VEGFA. To examine whether macrophages participated in the osteoblast precursor increase following osteoblast apoptosis, macrophage depletion models were employed. Depletion of macrophages via clodronate-liposomes and the CD169-diphtheria toxin receptor mouse model resulted in marked reduction in leptin receptor^+^ and osterix^+^ osteoblast precursors. Collectively, this work demonstrates the significance of osteoblast turnover via apoptosis and efferocytosis in postnatal bone formation. Importantly, it exposes the potential of targeting this mechanism to promote bone anabolism in the clinical setting.

## Introduction

Apoptosis occurs as part of normal development and tissue homeostasis. Among pertinent examples, apoptosis controls the number of neurons in the mammalian cortex^1^ and eliminates T cells with high affinity for self-peptides in the thymus.^2^ While it is estimated that 200–300 billion cells undergo apoptosis in the human body daily, dying cells are hardly observable in tissues and apoptotic materials do not usually elicit inflammation because of the impressive efficiency by which they are cleared from tissues termed “efferocytosis”. This process is performed by professional phagocytes among which are macrophages.^3^ Efferocytosis not only purges cellular corpses with inflammatory potential but also induces intracellular reprogramming that promotes pro-resolution signaling pathways.^4,5^ In the skeleton, the efferocytic mechanisms remain underexplored.

Osteoblasts mediate skeletal development via bone matrix deposition and mineralization. They differentiate from osteoprogenitors including those characterized by alpha-smooth muscle actin (αSMA)^6^ and leptin receptor (LepR)^7^ expression. The factors influencing recruitment or differentiation of osteoprogenitors remain incompletely understood but the latter requires the transcription factor osterix (OSX).^8^ Production of type I collagen (Col1a1) is one of the earliest events associated with osteoblastic differentiation and further development into mature osteoblasts is defined by expression of genes including osteocalcin (OCN).^9^ Some osteoblasts subsequently differentiate into osteocytes or become bone-lining cells but interestingly, the majority have been proposed to undergo apoptosis^10^ yet the relevance of this process in bone dynamics is unknown. The current general perception is that osteoblast apoptosis is detrimental to bone^11–13^ due to perceived impairment of bone formation and imbalance in remodeling.

Here, we aimed to examine the functional role of osteoblast apoptosis in postnatal bone development. Commonly used in vivo cell ablation strategies include the herpes simplex virus thymidine kinase gene (HSV-TK)/ganciclovir (GCV) approach^14^ and the transgenic expression of bacterial nitroreductase (NTR) or primate diphtheria toxin (DT) receptor (DTR).^15^ The HSV-TK/GCV and DTR models do not exclusively elicit apoptosis; they also induce pathological necrosis that cause pro-inflammatory responses.^16,17^ With NTR-mediated cell ablation, cell-permeable cytotoxins that can enter and kill adjacent non-targeted cells are produced, compromising selective targeting.^18^ We previously generated and characterized a mouse model with Cre-inducible caspase-9 (iCasp9) expression - the enzyme that initiates intrinsic apoptosis.^19^ In this model, treatment with a cell-permeable chemical compound (AP20187/AP) induces iCasp9 dimerization and thus, activation of the caspase-9-mediated apoptotic pathway, ultimately resulting in cell death. Apart from achieving selective stimulation of the apoptotic pathway, our model also utilizes a non-immunogenic dimerizer, avoiding the toxicities and/or production of neutralizing antibodies associated with the NTR ablation strategy or DT and GCV administration.^20,21^ This non-immunogenic feature allows prolonged efficacy in experiments with an extended time course. Using the OCNCre-iCasp9 mice, we investigated the process of osteoblast turnover via apoptosis and efferocytosis and revealed its importance in bone formation.

## Results

### AP treatment in OCNCre-iCasp9 mice induced OCN^+^ osteoblast apoptosis in the vertebra but not in the tibia

**Fig. 1:**
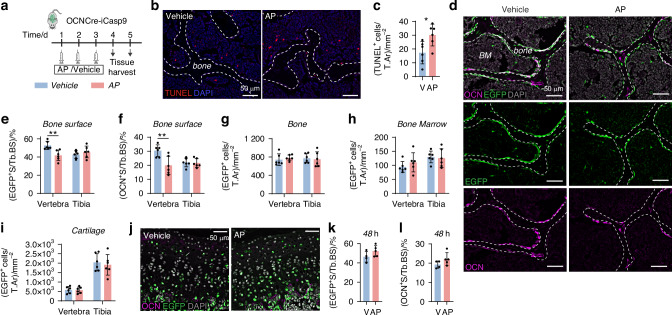
A three daily AP treatment in OCNcre-iCasp9 mice induced selective osteoblast apoptosis in the vertebra. **a** Schematic detailing the treatment regimen in 3-week-old OCNCre-iCasp9 mice and tissue harvest. **b** TUNEL staining with DAPI counterstain and (**c**) quantification of the number of TUNEL^+^ cells per tissue area (T.Ar) in the vertebrae of vehicle- or AP-treated OCNcre-iCasp9 mouse at 24 h post treatment. **d** Representative images showing distribution of EGFP and OCN expression with DAPI counterstain. BM bone marrow. Quantification in the vertebra and tibia of: (**e**) EGFP^+^ surface (S) per trabecular bone surface (Tb.BS), (**f**) OCN^+^ S per Tb.BS and (**g**) EGFP^+^ cell number per trabecular bone area (Tb.Ar) at 24 h post treatment. Number of EGFP^+^ cells in the (**h**) bone marrow or (**i**) cartilage expressed as per T.Ar at 24 h post treatment. **j** Images of EGFP and OCN expression in vertebral epiphyseal endplate with DAPI counterstain. Percentage of (**k**) EGFP^+^ S and (**l**) OCN^+^ S per Tb.BS at 48 h after the final injection. Statistical significance was determined using two-tailed unpaired *t*-test (**c**, **k**, **l**) or two-way ANOVA with Sidak’s multiple comparisons test (**e**–**i**). *P < 0.05; **P < 0.01. Error bars represent standard deviation. Each data point represents a single mouse. **b**–**j** *n* = 6 mice/group; (**k**, **l**) V *n* = 4 mice, AP *n* = 5 mice

We first determined an AP dose that can achieve selective apoptosis in OCN^+^ osteoblasts with the objective of stimulating apoptosis in a proportion of osteoblasts rather than completely ablating them. Augmented osteoblast apoptosis was achieved with a single 50 µg/g AP injection.^19^ Here, we tested whether half of this dose could also induce osteoblast apoptosis. One day after three daily treatments (Fig. 1a), TUNEL staining confirmed an increase in apoptotic signals in the vertebrae of 3-week-old AP-treated mice (Fig. 1b, c). In our iCasp9 mouse model, EGFP expression can be used to detect the iCasp9-expressing cells.^19^ Aligned with our previous finding, EGFP was detected in bone-lining osteoblasts as well as some osteocytes, bone marrow cells and chondrocytes (Fig. S1a). The short-term AP treatment, however, only induced reduction in EGFP^+^ (Figs. 1d, e and S1a) and OCN^+^ (Figs. 1d, f and S1a) cells on the bone surface of the vertebra without affecting the frequencies of EGFP^+^ osteocytes (Fig. 1g), marrow cells (Fig. 1h) and chondrocytes (Fig. 1i, j). In the tibia, the utilized AP treatment regimen had no impact on TUNEL (Fig. S1b), or EGFP- and OCN-expressing cells (Figs. 1e–i and S1c). The frequencies of EGFP^+^ and OCN^+^ osteoblasts remained unaltered even when a single dose of 50 µg/g of AP was used (Fig. S1d, e). Notably, the reduction in EGFP^+^ and OCN^+^ osteoblasts in the vertebra was no longer detectable at 48 h after the last AP injection (Figs. 1k, l and S1f). This demonstrates the transient effect of AP in inducing osteoblast apoptosis and indicates that the cells that had undergone apoptosis had been replaced by 48 h.

### Induction of osteoblast apoptosis stimulated macrophage efferocytosis

**Fig. 2:**
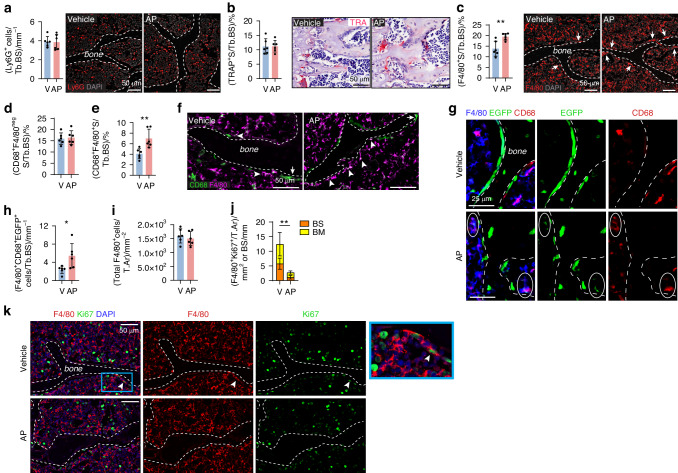
Osteoblast apoptosis in OCNCre-iCasp9 mice stimulated macrophage efferocytosis*.* **a** Quantification of the number of Ly6G^+^ cells directly associated with vertebral trabecular bone surface (Tb.BS) and representative images showing Ly6G expression with DAPI counterstain. **b** TRAP^+^ surface (S) per Tb.BS quantified using colorimetric TRAP assay counterstained with hematoxylin. **c** Quantification of percent F4/80^+^ S per Tb.BS and representative images of F4/80 staining with DAPI counterstain. Arrows indicate macrophages adjacent to bone. **d** Quantification of CD68^+^F4/80^neg^ S and (**e**) CD68^+^F4/80^+^ S per Tb.BS using (**f**) dual staining for CD68 and F4/80 expression. Arrowheads indicate cells with double positivity for CD68 and F4/80. Arrows indicate CD68^+^ cells negative for F4/80 expression. **g** Multiplex staining for F4/80, EGFP and CD68 expression used to quantify (**h**) F4/80^+^ cells that also expressed CD68 and EGFP. **i** Enumeration of the F4/80^+^ signals in the entire vertebral section expressed as number per tissue area (T.Ar). **j** Quantification of F4/80^+^Ki67^+^ cells in the entire vertebral section showing the proportion of signals on the bone surface (BS, mm) and in the bone marrow (BM, tissue area/T.Ar, mm^2^) and (**k**) representative images showing dual staining for Ki67 and F4/80 expression with DAPI counterstain. Arrowhead indicate a Ki67^+^F4/80^+^ cell. Statistical significance was determined using two-tailed unpaired *t*-tests or two-way ANOVA with Sidak’s multiple comparisons test. *P < 0.05; **P < 0.01. Error bars represent standard deviation. Each data point represents a single mouse. *n* = 6 mice/group

Efferocytosis was examined in the vertebra 24 h after the last AP injection. We previously provided evidence that osteal macrophages (osteomacs) can mediate the clearance of apoptotic osteoblasts.^19^ Imaging flow cytometry revealed that macrophages are fragmented during bone marrow disaggregation and fragmented remnants adhere to other cells, confounding ex vivo macrophage profiling via flow cytometry.^22^ Therefore, we opted for in situ analyses in the current study. The number of Ly6G^+^ neutrophils on bone surfaces was unchanged with osteoblast apoptosis stimulation (Fig. 2a). Similarly, TRAP^+^ osteoclast frequency remained unaltered (Fig. 2b). Examination of the pan-macrophage marker, F4/80, revealed an increase in macrophages associated with bone surfaces (i.e. osteomacs; Fig. 2c).

To extend characterization of the macrophages efferocytosing apoptotic osteoblasts, we examined other previously employed markers. Mac-2 has been used as a marker of efferocytic macrophages in response to thymocyte apoptosis.^4,23^ However, in the vertebra, Mac-2 is highly expressed by chondrocytes (Fig. S1g) and, therefore, was not used as an efferocytosis marker in this study. CD68 is a commonly used marker for phagocytes with its expression mainly localized in the endosomal/lysosomal compartment.^24^ As CD68 is expressed by both osteoclasts and macrophages, co-expression with F4/80 was used to differentiate these closely-related but distinct myeloid lineage cells.^25^ While CD68^+^F4/80^neg^ cells, many of which have typical osteoclast size and morphology, were unchanged with AP, CD68^+^F4/80^+^ osteomacs were significantly increased (Figs. 2d–f and S1h). Multiplex staining for F4/80, CD68 and EGFP expression (Fig. 2g) combined with an AI-driven quantification (Fig. S1i) confirmed that more F4/80^+^CD68^+^ osteomacs contained EGFP^+^ signals with AP treatment (Fig. 2h), indicative of increased efferocytosis. Similarly, F4/80^+^ cells that contained OCN^+^ remnants were significantly increased with AP (Fig. S1j, k).

Efferocytosis has been shown to induce proliferation in some macrophages.^4^ Despite the increase of osteomacs on the bone surface with AP treatment (Fig. 2c), the total number of F4/80^+^ cells in the vertebra was unchanged (Fig. 2i). Examination of Ki67 expression demonstrated that very few F4/80^+^ osteomacs proliferate near the trabecular bone of 3-week-old mouse vertebrae (Fig. 2j, k) at least under homeostatic condition. The total number of Ki67^+^F4/80^+^ signals in the bone was decreased with AP treatment (Fig. 2j, k).

### Long-term pulsed stimulation of osteoblast apoptosis increased vertebral bone formation

**Fig. 3:**
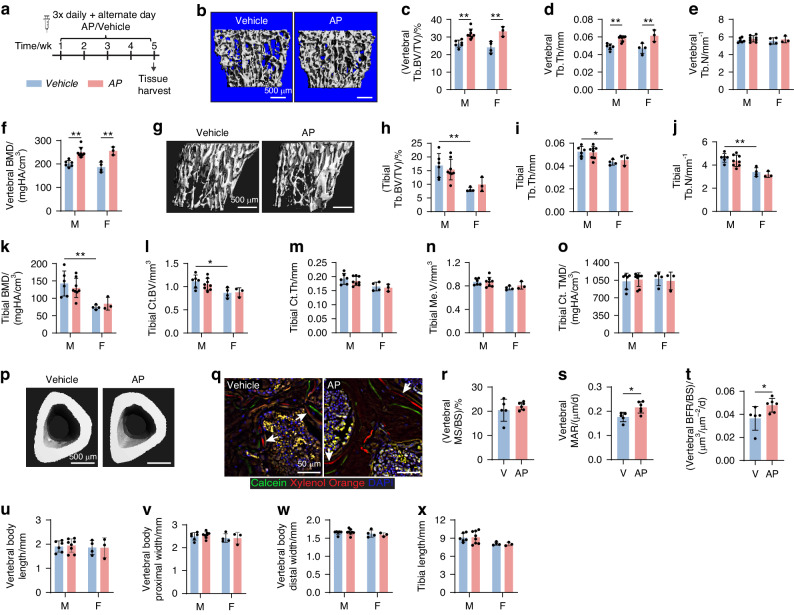
AP treatment for 5 weeks in OCNCre-iCasp9 mice increased vertebral bone formation*.* **a** Schematic detailing treatment regimen and timing of tissue harvest. **b** Representative micro-CT 3D reconstruction and analysis of the vertebrae showing trabecular (Tb) (**c**) bone volume (BV) per tissue volume (TV), (**d**) thickness (Th), (**e**) number (N) and (**f**) bone mineral density (BMD). Data from male (M) and female (F) mice are presented separately. **g** Representative micro-CT 3D reconstruction of the metaphyseal trabecular bone in the tibia. Micro-CT analysis showing Tb (**h**) BV/TV, (**i**) Th, (**j**) N, and (**k**) BMD. Micro-CT analysis of the cortical (Ct) bone showing (**l**) BV, (**m**) Th, (**n**) medulla volume (Me.V), (**o**) tissue mineral density (TMD) and (**p**) representative images with transverse view. **q** Dynamic bone labeling using calcein (7 days) and xylenol orange (2 days) in vertebral trabeculae. Sections were stained with DAPI to visualize nuclei. **r** Mineralizing surface per bone surface (MS/BS), (**s**) mineral apposition rate (MAR) and (**t**) bone formation rate per unit of bone surface (BFR/BS) quantified in the vertebral trabeculae using dynamic bone labeling (**q**). Assessment of vertebral body size showing height (**u**) and width of the proximal (**v**) or distal (**w**) bone ends. **x** Length of the tibiae measured from the growth plate to the tibiofibular junction. V vehicle. Statistical significance was determined using two-way ANOVA with Sidak’s multiple comparisons test (**c**–**f,** **h**–**o, u**–**x**) or two-tailed unpaired *t*-tests (**r**–**t**). *P < 0.05; **P < 0.01. Error bars represent standard deviation. Each data point represents a single mouse. **b**–**p, u**–**x** vehicle M *n* = 6 mice, AP M *n* = 8 mice, vehicle F *n* = 4 mice; AP F *n* = 3 mice; (**q**–**t**) vehicle *n* = 5 mice, AP *n* = 6 mice

The functional contribution of osteoblast apoptosis during postnatal bone development was next examined. Considering the transient nature of AP-induced osteoblast apoptosis (Fig. 1), we performed a long-term pulsed induction of osteoblast apoptosis by administering three daily AP/vehicle injections followed by every other day treatment for five weeks in 3-week-old male and female mice (Fig. 3a). This alternate day regimen would allow osteoblast frequency to return to baseline levels prior to the subsequent apoptosis induction (Fig. 1e, f, k, l). Micro-computed tomography (micro-CT) analysis (Fig. S2a) revealed a significant increase in vertebral fractional bone volume in AP-treated male and female mice compared to vehicle-treated controls (Fig. 3b, c). This increase was attributed to higher trabecular thickness (Fig. 3d) but not number (Fig. 3e). The vertebral trabecular bone mineral density (BMD) was also higher in the AP-treated groups (Fig. 3f). In the tibia (Fig. S2b), none of the trabecular (Fig. 3g–k) or cortical (Fig. 3l–p) bone parameters were altered by AP treatment. There were no sex differences detected in any of the vertebral bone parameters examined (Fig. 3c–f). In the tibia, reduced trabecular bone volume (Fig. 3h–j) and density (Fig. 3k), and cortical bone volume (Fig. 3l) were observed in females when compared to males at the age assessed (8 weeks at the time of harvest).

The vertebral trabecular bone accrual in response to AP treatment was further examined by dynamic bone labeling using calcein and xylenol orange administered at 7 and 2 days prior to harvest, respectively (Fig. 3q). As no sex differences in vertebral bone (Fig. 3c–f) were noted at this age, data from both sexes were pooled for subsequent histomorphometric and mechanical analyses. While mineralizing surface was similar between the groups (Fig. 3r), significant increases in mineral apposition rate (MAR, Fig. 3s) and bone formation rate (BFR, Fig. 3t) in vertebral trabeculae were noted with AP treatment. In the tibia, long-term AP treatment did not induce dynamic changes in the trabecular compartment (Fig. S2c–e). In the endocortical region, the mineralizing surface was unchanged (Fig. S2f). Although MAR was increased in this region (Fig. S2g), it was insufficient to drive a statistically significant increase in BFR (Fig. S2h). Although long-term administration of AP resulted in a significant increase in vertebral fractional bone volume, it did not affect the growth or size of this tissue (Fig. 3u–w), nor did it influence the length of the tibiae (Fig. 3x).

### Increased vertebral bone did not result in improved bone strength likely due to microporosity

**Fig. 4:**
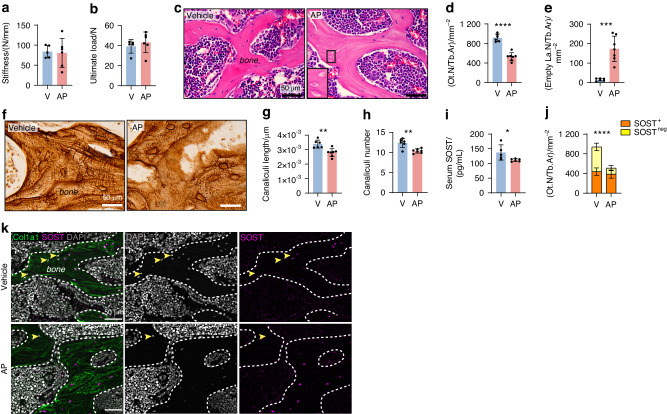
Long-term AP treatment in OCNCre-iCasp9 mice did not alter vertebral bone strength but disrupted the lacuno-canalicular network*.* Outcome of L5 vertebrae compression testing showing (**a**) stiffness and (**b**) ultimate load to failure. V vehicle. **c** H&E staining of the vertebra used to enumerate (**d**) osteocyte number (Ot.N) per trabecular bone area (Tb.Ar) and (**e**) empty lacunae number (La.N) per Tb.Ar. **f** Silver nitrate staining used to quantify the (**g**) average length of the canaliculi and (**h**) average number of canaliculi per osteocyte. **i** Levels of circulating sclerostin (SOST) at the experimental endpoint. **j** Proportion of SOST^+^ and SOST^−^ osteocytes in the trabecular bone quantified using (**k**) dual collagen type 1 (Col1a1) and SOST staining with DAPI counterstain. Arrowheads indicate the osteocytes negative for SOST staining. Statistical significance was determined using two-tailed unpaired *t*-tests or two-way ANOVA with Sidak’s multiple comparisons test. Error bars represent standard deviation. *P < 0.05; **P < 0.01; ***P < 0.001; ****P < 0.000 1. Each data point represents a single mouse. **a**, **b**, **i** V *n* = 5 mice, AP *n* = 6 mice; (**c**–**h**, **j**–**k**) V *n* = 6 mice, AP *n* = 7 mice

Compression testing of the L5 vertebrae showed that AP treatment did not improve bone stiffness (Fig. 4a) or ultimate load to failure (Fig. 4b) despite the significant increase in fractional bone volume and density (Fig. 3c, f). Examination of tissue sections (Fig. 4c) revealed a reduction in osteocyte number (Fig. 4d) and an increase in empty lacunae (Fig. 4e) within vertebral trabeculae. In the tibia, empty osteocyte lacunae were also increased although the total osteocyte number was unaltered (Fig. S2i–k). The canalicular network in the vertebra was further examined using silver nitrate staining (Fig. 4f). The remaining osteocytes in AP-treated mice were characterized by shorter (Fig. 4g) and less (Fig. 4h) canaliculi when compared to vehicle-treated controls.

Sclerostin is a protein primarily produced by osteocytes to inhibit bone formation.^26^ While the level of circulating sclerostin was significantly reduced following long-term AP treatment (Fig. 4i), the local sclerostin expression as demonstrated by the number of sclerostin^+^ osteocytes (Fig. 4j, k) was unchanged with the majority of the remaining osteocytes expressing sclerostin (Fig. 4j). The number of osteocytes and empty lacunae as well as serum sclerostin were not altered after short-term AP treatment (Fig. S2l–n).

### Long-term stimulation of osteoblast apoptosis was associated with increased osteoblast precursors

**Fig. 5:**
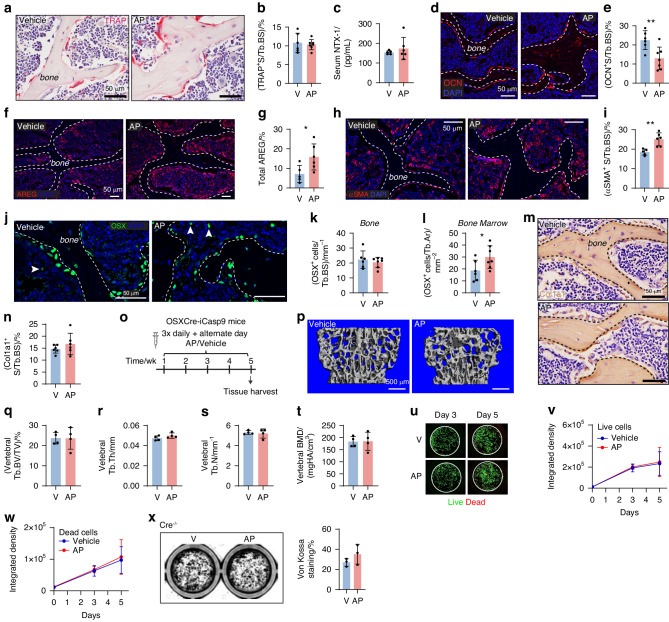
Long-term AP treatment in OCNCre-iCasp9 mice increased osteoblast precursors. **a**–**n** Treatment for 5 weeks in 3-week-old OCNCre-iCasp9 mice. **a** Colorimetric TRAP staining with hematoxylin counterstain in the vertebra of vehicle (V)- and AP-treated mice used to quantify (**b**) TRAP^+^ surface (S) per trabecular bone surface (Tb.BS). **c** Serum levels of N-Terminal Telopeptide of Type I Collagen (NTX-1) at the experimental endpoint. **d** Representative images of OCN staining with DAPI nuclear stain and (**e**) quantification of OCN^+^ S per Tb.BS. **f** Amphiregulin (AREG) expression with DAPI nuclear stain and (**g**) quantification of percent AREG^+^ staining in the entire vertebral tissue. **h** αSMA expression with DAPI nuclear stain. **i** Percent αSMA^+^ staining on the bone surface. **j** OSX expression with DAPI nuclear stain. Arrowheads indicate OSX^+^ cells in the bone marrow space. Number of osterix^+^ cells (**k**) on the Tb.BS and (**l**) in the bone marrow expressed as number per tissue area (T.Ar). **m** Collagen Type 1 (Col1a1) staining counterstained with hematoxylin and (**n**) quantification of percent Col1a1^+^ surface per Tb.BS. **o**–**t** Treatment for 5 weeks in 3-week-old OSXCre-iCasp9 mice of mixed gender. **o** Schematic detailing treatment regimen and timing of tissue harvest. **p** Micro-CT images of vehicle- or AP-treated OSXCre-iCasp9 mouse vertebra and analysis showing trabecular (Tb) (**q**) bone volume (BV) per tissue volume (TV), (**r**) thickness (Th), (**s**) number (N) and (**t**) bone mineral density (BMD). **u**–**w** Representative images (**u**) and frequencies of live (**v**) and dead (**w**) cells in BMSC culture isolated from OCNCre^−/−^ mice exposed to vehicle or AP. **x** Von Kossa staining of BMSCs isolated from OCNCre^−/−^ mice and treated with vehicle or AP for 12 days. Statistical significance was determined using two-tailed unpaired *t*-tests (**b**–**t**, **x**) or two-way ANOVA with Sidak’s multiple comparisons test (**v**, **w**). Error bars represent standard deviation. *P < 0.05; **P < 0.01. Each data point represents a single mouse. **a**, **b**, **d**, **e**, **j**–**l** V *n* = 6 mice, AP *n* = 7 mice; (**c**, **f**–**i**) V *n* = 5 mice, AP *n* = 6 mice; (**m**, **n**) *n* = 6 mice/group; (**o**–**t**) *n* = 4 mice/group; (**u**–**x**) *n* = 3 mice/group

We next investigated the cellular mechanism of the paradoxical bone accrual in response to osteoblast apoptosis. The frequency of TRAP^+^ osteoclasts on bone surfaces was unchanged following long-term AP-treatment (Fig. 5a, b). The levels of serum N-terminal telopeptide of type-1 collagen (NTX-1), a biomarker for bone resorption,^27^ were also similar between the groups at the experimental endpoint (Fig. 5c). Of note, even short-term AP treatment had no impact on serum NTX-1 levels (Fig. S2o). These indicated that osteoclastic resorptive activity was unaffected by short- or long-term AP administration, excluding it from the mechanism/s that contributed to the observed bone accrual. We did not examine C-terminal telopeptide of Type 1 collagen (CTX-1) since CTX-1 serum levels are influenced by food intake^28^ and the mice herein did not undergo a fasting state. There was a reduction in mature OCN^+^ osteoblasts (Fig. 5d, e) at the experimental endpoint, consistent with the impact of AP-induced apoptosis.

Amphiregulin (AREG) is a growth factor that can stimulate osteoblast precursor proliferation^29^ and promote their survival.^30^ Assessment of AREG expression in the vertebrae of OCNCre-iCasp9 mice treated long-term showed a marked increase with AP treatment (Fig. 5f, g). Staining for αSMA confirmed abundant expression in some vessels and weaker but positive expression in other cells residing within the bone marrow microenvironment (Fig. S3a). The frequency of αSMA^+^ cells on bone surfaces were significantly increased with osteoblast apoptosis stimulation (Fig. 5h, i).

OSX expression was largely associated with bone surfaces although some OSX^+^ cells were also present in the marrow space (Figs. 5j and S3b). While the number of OSX^+^ cells on the bone surface remained unaltered (Fig. 5j, k), OSX^+^ cells in the bone marrow were significantly increased with osteoblast apoptosis stimulation (Fig. 5j, l). The frequency of Col1a1^+^ cells lining the trabecular bone were also similar between the AP- and vehicle-treated groups (Fig. 5m, n). Given the reduction in mature OCN^+^ osteoblasts (Fig. 5d, e), the relative frequencies of OSX^+^ (Fig. 5k) and Col1a1^+^ (Fig. 5n) versus OCN^+^ cells on the bone surface indicates that more OSX^+^OCN^neg^ and Col1a1^+^OCN^neg^ pre- and/or immature osteoblasts were lining the bone at the experimental endpoint. We were unable to perform co-staining for these markers given the antibodies used were raised in similar species. Interestingly, a similar 5-week AP treatment regimen in OSXCre-iCasp9 mice (Fig. 5o) where OSX^+^ cells were targeted did not impact vertebral fractional bone volume (Fig. 5p, q), trabecular thickness (Fig. 5r), trabecular number (Fig. 5s) or BMD (Fig. 5t).

We also addressed whether the chemical dimerizer AP itself possessed osteoinductive actions that may have contributed to the observed osteoanabolic phenotype. BMSCs collected from OCN-Cre^−/−^ mice were used to assess cellular proliferation and mineral deposition. After 3 or 5 days of vehicle or AP treatment, neither live nor dead cell numbers were impacted by AP, suggesting no direct effect on BMSC proliferation (Fig. 5u–w). Similarly, no differences in Von Kossa staining of BMSCs treated with vehicle or AP for 12 days were observed (Fig. 5x), supporting that AP itself does not directly modulate osteogenic mineralization.

### Long-term induction of osteoblast apoptosis was associated with stimulation of macrophage efferocytosis and vasculature formation

**Fig. 6:**
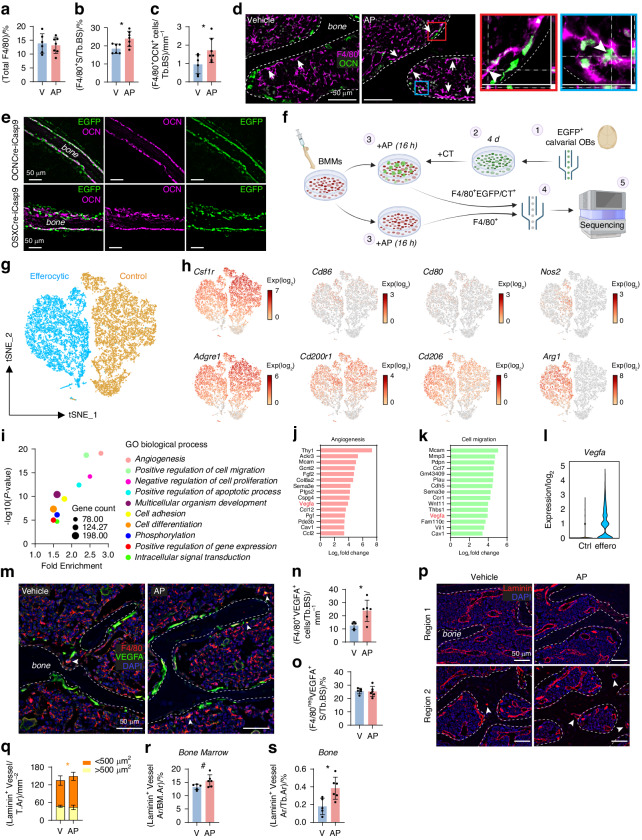
Long-term AP treatment in OCNCre-iCasp9 mice stimulated macrophage efferocytosis and vasculature formation*.* Quantification of (**a**) the total F4/80 staining in vertebral tissue and (**b**) F4/80^+^ surface (S) per trabecular bone surface (Tb.BS) after long-term AP or vehicle treatment in OCNCre-iCasp9 mice. **c** Number of F4/80^+^ cells containing OCN^+^ remnants per Tb.BS quantified using (**d**) sections stained for F4/80 and OCN expression. Arrows indicate macrophages associated with bone. Boxed regions include z-stack images showing intracellular OCN^+^ remnant (arrowhead) within a macrophage. **e** EGFP and OCN staining showing very similar expression in the calvaria of 1-week-old OCNCre-iCasp9 and OSXCre-iCasp9 mice. **f** Schematic summarizing the experimental flow from cell isolation to single-cell RNA sequencing. Schematic created with BioRender.com. CT Cell Tracker (CFSE). **g** tSNE plot showing clustering of 8 391 “efferocytic” macrophages and 10 036 “control” macrophages based on gene expression. **h** Dot plots showing expression (log_2_) of *Csf1r*, *Cd86*, *Cd80*, *Nos2*, *Adgre1*, *Cd200r1*, *Cd206* and *Arg1*. **i** Top 10 Gene Ontology (GO) biological process terms presented along with gene count, fold enrichment and statistical significance. List of top 15 upregulated genes within the GO term (**j**) “angiogenesis” and (**k**) “positive regulation of cell migration” ranked based on log_2_ fold-change. **l** Violin plot showing *Vegfa* expression in control (Ctrl) and efferocytic (Effero) macrophages. **m** Representative images showing VEGFA and F4/80 expression with DAPI counterstain. Arrowheads indicate some F4/80^+^VEGFA^+^ cells associated with bone. Quantification of (**n**) F4/80^+^VEGFA^+^ and (**o**) F4/80^neg^VEGFA^+^ cell numbers per Tb.BS. **p** Laminin expression with DAPI counterstain. Quantification of (**q**) the number of laminin^+^ vessels per tissue area (T.Ar) separated based on size. **r** Percent area (Ar) of laminin^+^ vessels per bone marrow area (BM.Ar). **s** Percent area of laminin^+^ vessels per trabecular bone area (Tb.Ar). Statistical significance was determined using two-tailed unpaired *t*-tests (**a**–**c**, **n**–**o**, **r**–**s**) or two-way ANOVA with Sidak’s multiple comparisons test (**q**). Error bars represent standard deviation. *P < 0.05; ^#^*P* = 0.067. Each data point represents a single mouse. **a–d** V *n* = 6 mice, AP *n* = 7 mice; (**e**–**l**) *n* = 3–4 mice/group; (**m**–**s**) V *n* = 5 mice, AP *n* = 6 mice

While there was no difference in the total F4/80^+^ staining in the vertebrae between the groups (Fig. 6a), F4/80^+^ osteomacs were significantly increased with long-term AP treatment (Fig. 6b). Many of these osteomacs contained OCN^+^ remnants (Fig. 6c, d), confirming continued efferocytosis stimulation until the experimental endpoint.

To identify other markers and biological processes associated with efferocytosis, single-cell RNA sequencing of in vitro generated efferocytic macrophages was performed. We examined macrophages generated in vitro (i.e. bone marrow-derived macrophages or BMMs) to avoid the fragmentation issue associated with ex vivo bone marrow disaggregation.^22^ Bone cells were isolated from the calvaria where EGFP (the marker of iCasp9-expressing cells) is largely expressed by OCN^+^ cells irrespective of the Cre driver strain (ie. OCNCre versus OSXCre, Fig. 6e and Fig. S4a). Efferocytic macrophages (F4/80^+^ and EGFP/Cell Trace^+^) were collected from BMMs co-cultured with AP-induced apoptotic bone cells and compared with “control” macrophages (F4/80^+^) cultured alone (Fig. 6f, Fig. S4b, c). Efferocytic macrophages exhibited a distinct transcriptomic signature (Fig. 6g) with 2881 genes upregulated compared to controls. We confirmed widespread expression of macrophage lineage genes *Csf1r* and *Adgre1* (gene encoding the F4/80 antigen) in both control and efferocytic macrophages (Fig. 6h). Examination of genes associated with M1-like pro-inflammatory (*Cd86*, *Cd80*, *Nos2*) versus M2-like anti-inflammatory (*Cd200r1*, *Cd206*, *Arg1*) macrophages^31^ showed distinctive expression of *Nos2* in a subset of efferocytic macrophages and a striking upregulation of *Arg1* in the entire efferocytic population (Fig. 6h). Other “M1” and “M2” markers had variable expression in both populations (Fig. 6h).

Using Gene Ontology (GO) analysis of upregulated genes, we identified the top enriched biological processes including “angiogenesis” and “positive regulation of cell migration” (Fig. 6i). The latter (cell migration) is particularly interesting given our finding that macrophages are likely recruited to the bone surface in response to osteoblast apoptosis rather than local proliferation (Fig. 2i–k). Aligned with this, macrophage efferocytosis of apoptotic bone cells in vitro led to expression of chemotaxis-associated genes including *Ccr1*, *Ccl2*, *Ccl7* and *Ccl12* (Fig. S4d). *Vegfa* was a common upregulated gene present in the top 15 genes associated with “angiogenesis” and “positive regulation of cell migration” (Fig. 6j–l). VEGFA is known to be expressed by macrophages to stimulate angiogenesis but it can also serve as their chemotactic factor.^32^

VEGFA expression by efferocytic macrophages was further confirmed in vivo by examining this protein in the vertebra of OCNCre-iCasp9 mice following long-term AP treatment (Figs. 6m and S5a). Dual F4/80 and VEGFA staining confirmed an increase in F4/80^+^VEGFA^+^ osteomacs with long-term AP administration (Fig. 6m, n). Although osteoclasts and osteoblasts can express VEGFA (Fig. 6m), AP had no impact on the frequency of F4/80^neg^VEGFA^+^ bone-associated cells (Fig. 6o).

We also examined vasculature formation since single-cell RNA sequencing analysis identified “angiogenesis” as the top enriched biological process based on fold enrichment (Fig. 6i). There was an increase in the number of small laminin^+^ vessels with a cross-sectional area of less than 500 µm^2^ in the vertebral tissue of mice that received osteoblast apoptosis stimulation (Fig. 6p, q, Fig. S5b). In the bone marrow, there was a trend towards increase in the total area of laminin^+^ vessels (Fig. 6p, r) while in the bone, the total area of these vessels was significantly increased (Fig. 6p, s). Collectively, these results suggested that long-term AP treatment resulted in the formation of smaller vessels.

### Macrophage depletion resulted in reduced osteoblast precursors

**Fig. 7:**
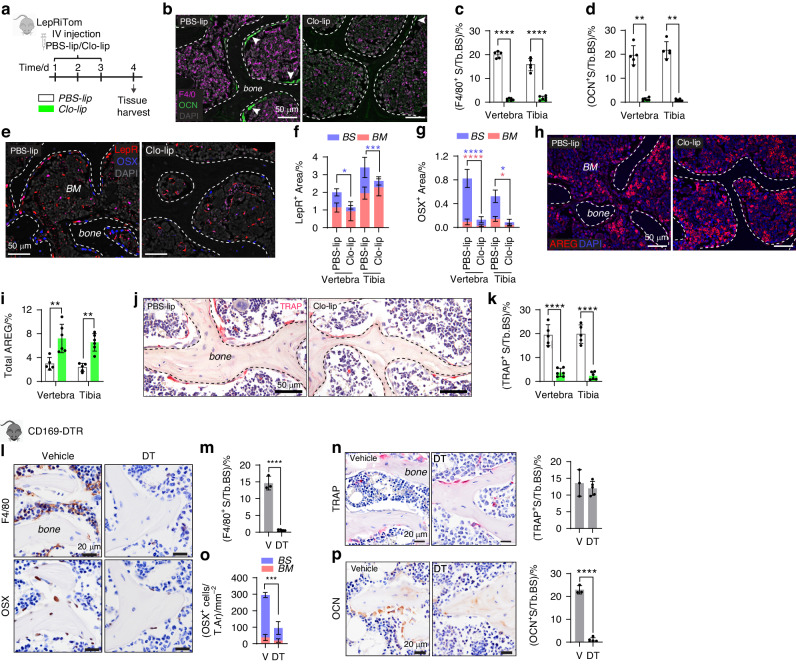
Macrophage depletion reduced osteoblast precursors. **a** Schematic detailing the clodronate- or PBS-liposome treatment regimen in LepRiTom mice and tissue harvest. **b** Dual F4/80 and OCN staining with DAPI nuclear stain in the vertebra. Arrowheads indicate OCN^+^ staining on the bone surface. Quantification of (**c**) F4/80^+^ or (**d**) OCN^+^ surface (S) per trabecular bone surface (Tb.BS) in the vertebra and tibia. **e** LepR and OSX staining in the vertebra with DAPI counterstain. Area of (**f**) LepR^+^ or (**g**) OSX^+^ staining on the bone surface (BS) or bone marrow (BM) expressed as percentage of the total vertebral section. Significance of each group presented in the same color as the corresponding bar. **h** Amphiregulin (AREG) expression in the vertebra and (**i**) quantification of percent AREG^+^ staining per total area within the tissues examined. **j** TRAP staining and (**k**) quantification of percent TRAP^+^ S per Tb.BS. **l**–**p** Histomorphometric analysis of the metaphyseal trabecular bone of CD169-DTR mouse tibia following vehicle (V) or DT treatment. **l** Near serial sections stained with F4/80 or OSX. Quantification of percent (**m**) F4/80^+^ and (**n**) TRAP^+^ S per Tb.BS with representative images. **o** Total number of OSX^+^ cells per area of tissue (T.Ar) examined with proportion on the bone surface (blue) versus marrow (red). **p** OCN expression and quantification of OCN^+^ surface expressed as a percentage of the Tb.BS. Statistical significance was determined using two-way ANOVA with Tukey’s multiple comparisons test (**c**, **f**, **g**, **i**, **k**), unpaired multiple Mann–Whitney *t*-test (**d**) or two-tailed unpaired *t*-tests (**m**–**p**). Error bars represent standard deviation. *P < 0.05; **P < 0.01; ***P < 0.001; ****P < 0.000 1. Each data point represents a single mouse. **b**–**k** PBS-lip *n* = 5 mice, clo-lip *n* = 6 mice; (**l**–**p**) V *n* = 3 mice, DT *n* = 5 mice

Thus far, our results demonstrated that osteoblast apoptosis stimulated macrophage efferocytosis, increased osteoblast precursors and culminated in bone accrual. We assessed whether macrophages themselves contributed to the observed increase in osteoblast precursor number by utilizing macrophage ablation strategies. Of note, previous studies demonstrated a concomitant loss of osteoblasts when macrophages are depleted,^25,33^ however, whether this loss was due to a preceding impact on osteoblast precursors is unknown. Fate mapping of Lepr-Cre-expressing mesenchymal cells in vivo shows that they are the primary source of osteoblasts in adult bone.^7,34^ We examined the impact of clodronate-liposome (clo-lip)-induced macrophage depletion on LepR^+^ cells. A three daily clo-lip administration in LepRiTom mice (tdTomato-tagged LepR^+^ cells^35^) resulted in almost complete loss of osteomacs in both the vertebra and tibia (Fig. 7a–c). This loss was associated with a significant reduction in OCN^+^ osteoblasts (Fig. 7b, d). In control tissues, LepR expression was abundant in both the bone marrow and on the bone surface (Figs. 7e and S5c, d). Co-staining for OCN expression showed a population of LepR^+^ cells on the bone surface that were negative for OCN (Fig. S5c), likely representing an undifferentiated precursor population. Clo-lip treatment resulted in the reduction of LepR^+^ cells on the bone surface but not in the marrow (Fig. 7f), indicative of reduced bone-associated osteoblast precursors. Some cells on the bone surface were positive for both LepR and OSX, while some were only positive for either LepR or OSX (Fig. S5d), likely reflecting osteoblast lineage cells at various differentiation stages. OSX^+^ cells in the bone marrow and bone surface were also reduced after clo-lip administration (Fig. 7g). Interestingly, AREG was significantly increased in response to clo-lip treatment (Fig. 7h, i) suggesting that the reduction in osteoblast lineage was not due to the depletion of this growth factor.

We found that clo-lip administration also reduced TRAP^+^ osteoclasts (Fig. 7j, k). This was not surprising as clo-lip also targets osteoclasts because they are phagocytic.^36^ Therefore, to delineate the contribution of macrophages versus osteoclasts in regulating osteoblast precursor number, we used the CD169-DTR model, which can selectively target CD169^+^ macrophages such as osteomacs without affecting osteoclast frequency or activity.^25^ Indeed, DT treatment depleted osteomacs (Fig. 7l, m) without affecting osteoclast frequency (Fig. 7n). A similar reduction in OSX^+^ cells was observed in this model (Fig. 7l, o) which also resulted in osteoblast loss (Fig. 7p), further supporting the importance of macrophages in regulating osteoblast precursor number.

## Discussion

The contribution of apoptosis and efferocytosis in bone dynamics remain underexplored despite over two decades of recognition that apoptosis is a main fate of osteoblasts.^10^ The current study took advantage of the transient and inducible nature of our iCasp9 mouse model that enabled induction of controlled osteoblast apoptosis to examine its importance during postnatal bone development. Using this model, we provided in vivo functional evidence that osteoblast turnover via apoptosis involves osteomac efferocytosis and is an important mediator of postnatal bone formation.

Osteoblast apoptosis has been acknowledged as a contributing mechanism in several skeletal pathologies.^13^ Inhibition of osteoblast apoptosis has been implicated in the anabolic actions of parathyroid hormone,^37^ bisphosphonates^38^ and the calcitonin family^39^ while bone loss induced by aging and glucocorticoid treatment is, at least in part, caused by osteoblast death.^13^ Consequently, the general view of augmented osteoblast apoptosis is that it is detrimental to bone health. Therefore, our finding demonstrating that stimulation of osteoblast apoptosis can be anabolic may seem paradoxical. It is important to note that the titrated AP dose herein did not deplete the bone-forming cells rather it induced apoptosis in less than 50% of mature OCN-expressing osteoblasts. Furthermore, the alternate day administration of the dimerizer allowed recovery of mature osteoblasts to baseline level prior to the next apoptosis trigger. A previous study using the DTR approach to ablate OCN-expressing cells in mice every other day for two weeks revealed sexually dimorphic tibial phenotypes: decrease in female Tb.BV/TV, Tb.Th and BMD while unchanged in male Tb.BV/TV and BMD with increased Tb.Th.^40^ Another study using similar DTR model but with daily OCN^+^ cell ablation claimed inhibition of bone formation based solely on mouse size and the genders were not specified.^41^ Both DTR studies only characterized long bones whereas the bone accrual in our iCasp9 model occurred in the vertebrae of both genders. Aligned with our observations, however, neither study detected changes in osteoclast number or activity when targeting OCN^+^ cells. Fairfield et al. reported sudden death in 13.7% of DT-treated animals,^40^ which was likely due to DT-induced toxicity. In our study, no adverse events were observed with AP administration, even after five weeks of treatment. A limitation in both studies and ours was the inability to determine the anabolic activity of the remaining osteoblasts which could have also impacted the overall bone accrual.

While it is widely recognized that maintenance of osteoblast frequency requires constant differentiation from skeletal stem cells, the regulatory mechanisms underlying osteoblast renewal remain poorly understood. Our findings suggest that osteoblast apoptosis is an inducer of this process. Although stimulation of OCN^+^ cell death reduced mature osteoblasts, earlier lineage markers were increased in response. Furthermore, the bone accrual in OCNCre-iCasp9 mice was not mimicked in the OSXCre-iCasp9 model, which also targets earlier osteolineage cells,^34^ supporting that precursor recruitment or differentiation was necessary for the observed osteoanabolic phenotype. Overexpression of apoptosis inhibitors, BCL2^42^ and BCLXL,^43^ using the *Col1a1* promoter also modulated osteoblast differentiation although the results were markedly different phenotypes. Osteoblast differentiation was inhibited with BCL2 overexpression while promoted when BCLXL was targeted. BCLXL overexpression in *Col1a1*-expressing cells was also characterized by increased bone mass.^43^ It should be noted, however, that while this study found reduced TUNEL expression with BCLXL overexpression, the total density of osteoblasts in vivo was unchanged. Notable dissimilarities between this model and ours include the different promoters used and the constitutive versus inducible nature of the systems.

Osteomacs are the specialized bone-resident macrophages necessary for maintaining bone homeostasis and promoting fracture repair^25,33,44,45^; however, the specific mechanisms underlying their contributions in bone dynamics are largely unknown. Our findings implicate osteomacs and their archetypal efferocytic function in osteoblast turnover. We showed that efferocytosis by osteomacs is stimulated following short- and long-term induction of osteoblast apoptosis. Since Ki67 expression was reduced in efferocytic osteomacs, the increase in their number was likely through macrophage recruitment rather than proliferation. Although efferocytosis can induce macrophage proliferation,^4^ this process was demonstrated in response to high apoptotic cell burden (i.e. 5:1 apoptotic-macrophage ratio). In our study, apoptosis was induced only in a fraction of osteoblasts, so the frequency of local macrophages were likely sufficient. Osteomacs are highly enriched at sites of bone modeling and remodeling, and they often form a barrier-like structure over osteoblasts.^33,46^ They also support osteoclast activity via sequestration of resorption by-products.^47^ Therefore, osteomac involvement in osteoblast turnover via facilitating their clearance was not surprising and likely achieves compartmentalization of the dynamic bone sites as proposed previously,^46,48^ preventing leakage of immunogenic cellular corpses into the haematopoietic bone marrow, and vice versa. In terms of the mechanism, vesicles from apoptotic osteoblasts have been shown to stimulate macrophage migration via the activation of SIRT6-mediated chemotaxis.^49^ Furthermore, the actual efferocytic process is also modulated by SIRT6 via targeting the CD47-SIRPα checkpoint.^49^ Regarding activation state, efferocytic macrophages are generally considered to possess an M2-like phenotype.^50^ Our results showed that in response to bone cell apoptosis, efferocytic macrophages do not fully satisfy either the M1 or M2 classification. This is aligned with what many have argued that macrophages cannot be confined in an overly simplistic dichotomous model.^51–56^

Apart from the “clean-up” function of osteomacs, our findings also provide evidence of their participation in regulating osteoblast precursor recruitment or differentiation. We found that the process of macrophage efferocytosing apoptotic osteoblasts induces expression of factors with osteoinductive potential such as VEGFA.^57^ Additionally, loss of osteomacs resulted in reduced osteoblast precursors. The two macrophage depletion models used do not directly ablate osteoblasts since they are neither professional phagocytes nor CD169-expressing cells.^25^ In fact, in vitro, liposomal clodronate has been shown to stimulate osteoblast differentiation.^58^ Therefore, the observed osteoblast reduction herein was an indirect effect likely caused by impaired replenishment from precursors. In vitro studies have demonstrated that macrophages promote the osteogenic differentiation of multi-potent stromal cells/mesenchymal progenitors.^59–63^ Moreover, efferocytosis of apoptotic bone cells by macrophages leads to secretion of TGF-β1,^64^ which is directly involved in osteoblast differentiation.^65,66^ Therefore, a plausible hypothesis is that osteomac efferocytosis triggers a paracrine loop via production of factors that attract osteoblast precursors thereby sustaining bone formation activity. In support of the pro-anabolic role of macrophage efferocytosis in bone, Xu et al. recently demonstrated that enhancing macrophage efferocytosis of apoptotic osteoblasts in aged mice can mitigate the age-associated bone loss.^49^ We were unable to directly target osteomac efferocytosis in the current study due to the absence of strategies to achieve this. This technical limitation is an ongoing challenge in efferocytosis research complicated by the redundancy of currently identified receptors.^67^ For example, MERTK and TYRO3, which are well-studied efferocytic macrophage receptors,^68^ also have osteoblast-specific functions.^69^ Identifying the specific receptor/s for efferocytic osteomacs is essential for developing approaches that could provide unequivocal evidence for the role of efferocytosis in bone.

Bone accrual did not translate to improved bone strength likely due to increased micro-porosity from empty osteocyte lacunae as porosity influences bone mechanical strength.^70^ Overexpression of the apoptosis inhibitor BCL2 in osteoblasts also eventually caused osteocyte apoptosis,^42^ suggesting that alteration of osteoblast apoptotic mechanisms has direct impact on osteocyte survival. However, in our study, the reduction in osteocytes is arguably an effect of OCNCre limitation, which can also target osteocytes.^19,71^ This limitation is also shared by other widely employed “osteoblast-targeted” models including Col1a1Cre and Runx2Cre^72^ and to date, there is no Cre mouse line that solely targets osteoblasts. It is also worth noting that this limitation suggests the possibility that certain osteoblast precursors might have been affected, potentially contributing to the observed reduction in osteoblasts. However, the potential impact on osteoblast precursors is less likely to have caused the increased empty osteocyte lacunae. Short-term AP administration had no impact on the frequencies of osteocytes or empty lacunae. This indicates that the osteocyte lacuno-canalicular network changes following long-term treatment is gradual and additive, becoming significant only at the later time point likely because dead osteocytes are not as replaceable as osteoblasts which are directly accessible to the osteoprogenitors residing in the bone marrow.

The level of circulating sclerostin was reduced following long-term AP treatment; however, this was unlikely a mechanism of increased bone formation given bone accrual was not systemic. Serum sclerostin does not always reflect the local expression level as demonstrated herein and by others in both humans^73^ and mice.^74^ Moreover, while sclerostin is a well-known inhibitor of bone formation,^75^ a positive correlation of serum sclerostin levels with BMD or bone mineral content has been observed in numerous clinical studies.^76–79^ The relevance of serum sclerostin reduction in our study is unclear but a possible explanation is that sclerostin is retained locally to limit further increases in vertebral bone mass that might be physiologically detrimental to mice as this could displace the marrow. Alternatively, it may represent changes in sclerostin expression in other tissues not examined herein.

Intriguingly, the anabolic phenotype was only observed in the vertebra but not in the tibia. We did not detect changes in osteoblast apoptosis or macrophage efferocytosis in the tibia following either short- or long-term treatment. A potential reason could be variability in the regulation of anti-apoptotic mechanisms in these bones. While addressing this theory is beyond the scope of the current study, there is evidence that different tissues can have different anti-apoptotic responses to insults or treatments such as glucocorticoids.^80^ Another probable explanation is the presence of a vertebral skeletal stem cell population distinct from long bone stem cells^81^ which may be differently stimulated by osteoblast apoptosis. Another intriguing observation was the increased AREG in the macrophage depletion model. A potential explanation is that the reduction in osteoblast precursors and their differentiation resulted in decreased protein uptake, and thus increased the bioavailable AREG in the tissue.

In summary, our study demonstrates the importance of osteoblast turnover via apoptosis and osteomac efferocytosis in promoting bone formation. This work offers a perspective shift regarding osteoblast apoptosis, revealing that it is a crucial biological process for anabolism at least during early postnatal development. Further studies to unravel the critical signaling mechanisms linking osteoblast apoptosis to their renewal and how this pathway may be altered in disease could lead to identification of novel therapeutic interventions.

## Materials and methods

### Experimental animals

Animal experiments were approved by the University of Michigan (UM) Institute for Animal Care and Use Committee following the NIH Guide for the Care and Use of Laboratory Animals or by The University of Queensland Health Sciences Ethics Committee performed in accordance with the Australian Code of Practice for the Care and Use of Animals for Scientific Purposes. Homozygous iCasp9^+/+^ mice were generated as previously prescribed^19^ and bred with Osteocalcin (OCN)-Cre (B6.FVB-Tg(BGLAP-cre)1Clem/J; Jackson Laboratory, Bar Harbor, ME, USA) or Osterix (OSX)-Cre mice (B6.Cg-Tg(Sp7-tTA,tetO-EGFP/cre)1Amc/J; bred in-house) generating OCNCre^+/−^iCasp9^+/+^ (OCNCre-iCasp9) and OSXCre^+/−^iCasp9^+/+^ (OSXCre-iCasp9) mice, respectively. Leptin receptor (LepR) Tdtomato tagged mice were maintained by breeding LepR^tm2(cre)Rck+/-^ mice (LepR^+/−^)^82^ with Gt(ROSA)26Sor^tm(CAG-tdTomato)Hz+/+^ mice (Tom^+/+^)^83^ to generate LepRCre^+/−^Tom^+/−^ (LepRiTom) mice used for experimentation.^35^ CD169^+/DTR^ mice (Siglec1^tm1(HBEGF)Mtka^) called “CD169-DTR” here were originally sourced from Bio Resource Centre (Yokohama, Kanagawa, Japan). C57Bl/6J mice were maintained in-house. Both male and female mice were utilized. All animals were housed in 12 h light/dark cycle and provided free access to food and water.

### AP treatment and tissue collection

BMSCs were isolated and expanded from 5-week-old OCN-Cre^−/−^ mouse long bones.^84^ Passage 1 cells were plated overnight, then incubated with vehicle or AP (250 nmol/L). Live and dead cells within the culture were examined using the Live/Dead Cell Imaging Kit (Thermo Fisher Scientific, Waltham, MA, USA) at day 0 (untreated) and after 3 and 5 days with vehicle/AP. BMSCs were treated every 2–3 days with 50 μg/mL ascorbic acid and 10 mmol/L β-glycerophosphate along with vehicle/AP treatment. Mineralization was assessed at day 12 using Von Kossa staining as described previously.^84^ All in vitro images were taken through the Leica THUNDER imaging system (Leica Microsystem, ZQQ, DE) and analyzed using ImageJ (National Institutes of Health, NIH, Bethesda, MD, USA).

AP (Takara Bio, San Jose, CA, USA) and vehicle solution was prepared and administered intraperitoneally at 25 µg/g weight to 3-4-week-old OCNCre-iCasp9 mice or OSXCre-iCasp9 mice as previously described.^19^ Similar composition without AP was used as “vehicle”. Mice were randomly allocated to “AP” or “vehicle” groups and treated short-term (three daily injections) or long-term (three daily injections followed by every other day treatment for 5 weeks). Tissues were collected at 24 or 48 h after the final injection. In total, four independent experiments were conducted with sample sizes in each experiment dependent on litter sizes. When mixed sexes were used, as specified in the text, males and females were randomly and equivalently allocated to either vehicle or AP groups.

Blood collected from anesthetized mice via cardiac exsanguination was placed into a serum separator tube (Sai Infusion Technologies, Lake Villa, IL, USA). The fifth lumbar (L5) vertebra were frozen^85^ until mechanical testing. L1-L4 vertebrae and tibiae were fixed in 4% paraformaldehyde for 72 h and transferred to 70% ethanol for micro-computed tomography (micro-CT). Following micro-CT, bones were either processed for plastic embedding or decalcified and paraffin embedded. While tissues for paraffin-embedding were collected from all cohorts, tissues for mechanical testing and plastic processing were only collected in some cohorts given the destructive nature of these analyses on tissues.

### Macrophage depletion models

Two widely used macrophage depletion models were utilized: clodronate-liposomes (clo-lip) and the CD169-DTR mouse model. For both models, adult mice at ~12-week-old were used and were randomly allocated to treatment and control groups. Three daily intravenous injections of clo-lip or phosphate-buffered saline (PBS)–liposome (PBS-lip) (Liposoma BV, Amsterdam, The Netherlands) were administered to LepRiTom mice and tissues were collected 24 h after the last injection. For CD169-DTR experiments, the contralateral limbs of mice from a previously published experiment^25^ were examined. In brief, vehicle (0.9% sodium chloride) or diphtheria toxin (DT; MBL International Corporation, MA, USA) were administered at 10 μg/kg body weight intraperitoneally for 4 consecutive days and tissues were harvested 24 h after the last injection. Bones were decalcified and processed for paraffin embedding.

### Dynamic Histomorphometry

Calcein (30 mg/kg) and xylenol orange (90 mg/kg) were prepared in 2% sodium bicarbonate/PBS and administered subcutaneously at 7 or 2 days prior to harvest, respectively. Sections (5 µm) from plastic-embedded tissues were collected. Plastic was removed using routine protocol before mounting with Prolong Gold mounting medium with DAPI (Thermo Fisher Scientific). Slides were imaged using the Leica THUNDER imaging system (Leica Microsystem). The mineralizing surface, mineral apposition rate (MAR) and bone formation rate (BFR) were calculated in 200X tiled images according to the standardized nomenclature.^86^

### Enzyme-linked immunoassay (ELISA)

Blood was allowed to clot at room temperature and centrifuged at 5 000 rpm for 10 min. Serum was collected and stored at −80 °C until assay. Serum levels of N-terminal telopeptide of Type-1 collagen (NTX-1, Elabscience, Houston, TX) and sclerostin (R&D systems) were measured by ELISA according to the manufacturer’s protocol. Absorbance measurements were collected using Biochrom EZ Read 400 Microplate Reader (Biochrom US Inc, Holliston, MA, USA).

### Micro-CT and mechanical characterization

Fixed bones were scanned using Scanco’s µCT100 (Scanco Medical, Bassersdorf, Switzerland). The scan settings were standardized to 70 kVp and 114 µA, and the filter used was a 0.5 mm aluminum. The specimens were scanned over 180° rotation with 500 ms integration time. Analysis was performed blindly using the manufacturer’s evaluation software, and global thresholds of 180–1 000 for trabeculae and 280–1 000 for cortices were used to segment bone from non-bone. The trabeculae in the vertebrae, and trabeculae and cortices in the tibiae were examined in the regions specified in Fig. S2a, b. Vertebral bodies were assessed for the quantification of bone size which was measured from the top to the bottom of epiphyses. The width of this bone tissue was measured on both the proximal and distal ends. For the tibiae, since some samples exhibited broken distal ends, bone length was examined from the growth plate to the tibiofibular junction. Compression testing was chosen to determine mechanical properties of the vertebrae and conducted as previously described.^87^

### Immunofluorescence, immunohistochemistry and histological staining

Paraffin sections (5 µm) were collected and immunofluorescence (IF) or immunohistochemistry (IHC) were performed as previously described.^19^ Primary and secondary antibody information including catalog number, dilution, incubation period and antigen retrieval procedure are provided in Table S2. Slides were mounted using Prolong Gold with DAPI (Thermo Fisher Scientific) for IF or Histomount (Life Science Products Inc., Frederick, CO, USA) for IHC and standard histology. Colorimetric TRAP^88^ and silver nitrate^89^ staining were performed as described previously. Hematoxylin and Eosin (H&E) staining was performed using routine protocol. Slides were imaged using the Leica THUNDER microscope (Leica Microsystems) or VS120 slide scanner (Olympus, TYO, JP). Representative images were taken at 200X, 400X or 630X.

### Histomorphometry

Histomorphometric analyses were performed blindly on ImageJ (NIH) or by an automated approach using the AIVIA software (Leica Microsystem). The regions analyzed in the vertebra and tibia are indicated in Fig. S2a, b. ImageJ was used to quantify the lengths of OCN-, TRAP-, F4/80-, CD68-, αSMA- and Col1a1-positive staining associated with bone and expressed as a percentage of trabecular bone surface. The different subpopulations of EGFP^+^ cells (i.e. osteocytes, chondrocytes, BMSC and osteoblasts) were quantified as previously described.^19^ All quantifications related to laminin and the lacuno-canalicular network were performed on ImageJ. The canaliculi of 20 osteocytes per section were examined and the average values were collected.

The AIVIA software, which automatically separates fluorescent signals in red/blue/green (RGB) channels, was used to analyze the number of F4/80^+^CD68^+^EGFP^+^ signals. For the analysis, F4/80 was pseudo-colored as blue, CD68 as red and EGFP as green (Fig. S1e). Similar color thresholds were applied across all samples. The software was trained to detect cells where F4/80 expression overlapped with CD68 and EGFP staining. The software automatically detected the triple positive cells with high precision (Fig. S1e). Only signals associated with the bone were included in the quantification. A similar approach in AIVIA was used to detect Ki67^+^F4/80^+^ cells, VEGFA^+^F4/80^+^ cells (Fig. S3e), AREG^+^ staining, TUNEL^+^ signals, LepR^+^ cells and fluorescent osterix (OSX).

### Cell sorting and Single-Cell Library Preparation, RNA Sequencing and Gene Ontology Analysis

Bone cells were isolated from the calvaria^84^ because the expression patterns of EGFP and OCN were almost completely overlapping in this bone tissue likely due to the absence of chondrocytes and fewer bone marrow cells which have subsets that exhibited EGFP expression in other bones (Fig. 1). 1-week-old OSXCre-iCasp9 mouse calvaria (*n* = 4) which have comparable EGFP and OCN expression to OCNCre-iCasp9 mouse calvaria (Fig. 6e) was utilized. iCasp9-expressing calvarial bone cells were sorted (BD FACSAriaTM IIu cell sorting cytometer, BD Biosciences, San Jose, CA, USA) based on EGFP expression and expanded in culture^84^ (Fig. 6f). EGFP gating was established using cells from Cre-negative mouse calvaria that had undergone a similar isolation procedure (Fig. S4a). After expansion, CellTrace™ CFSE (CT; Thermo Fisher Scientific) which has similar excitation-emission spectra as EGFP was added into the bone cell culture to ensure sufficient intracellular fluorescent labeling needed for sorting.

Bone marrow-derived macrophages (BMMs) were generated from C57BL/6J mouse (*n* = 3) femora and tibiae.^90^ BMMs were cultured alone (*n* = 3 individual cultures) or with apoptotic bone cells at 1:2 macrophage-apoptotic cell ratio in the presence of 1 μmol/L AP (*n* = 3 individual cultures). After 16 h, the cells from 3 monocultures and cells from 3 co-cultures were pooled on separate tubes and stained with anti-F4/80-APC antibody (Abcam, Cambridge, MA, USA) for 30 min on ice. “Efferocytic” macrophages were sorted from the co-culture based on double positivity for F4/80 and EGFP/CFSE while “control” macrophages (F4/80^+^ cells) were sorted from the monoculture (Fig. S4b, c).

The single-cell RNA sequencing (scRNA-Seq) libraries were prepared at the University of Michigan Advanced Genomics Core using the Chromium Next GEM Single Cell 3′ Kit v3.1 (10X Genomics, Pleasanton, CA, USA) as per the manufacturer’s protocol. Cell suspensions were diluted to target a recovery of 10 000 cells per sample. The libraries were quality-controlled using TapeStation 4200 (Agilent Technologies, Santa Clara, CA, USA) before sequencing. The libraries were sequenced at a depth of 30 000 reads per cell in NovaSeq 6000 (Illumina, San Diego, CA, USA) using the following run configuration: Read 1–28 cycles; Index 1–10 cycles; Index 2–10 cycles; Read 2–150 cycles.

The scRNA-Seq data were processed using the CellRanger software suite v3.0.0 (10X Genomics). CellRanger was used to trim and analyze the raw data to the appropriate read lengths. The FASTQ files from each sample were mapped to the mouse genome mm10, and gene counts were obtained using CellRanger and STAR aligner.^91^ The barcode-gene matrices were further analyzed using CellRanger Aggregate and Loupe Browser 6.0 (10× Genomics) to extract the upregulated genes in efferocytic macrophages compared to controls. Functional annotation analysis of significantly upregulated genes (*P* < 0.05) were conducted using the Database for Annotation, Visualization, and Integrated Discovery (DAVID) tool.^92,93^ The GO terms (Biological Process) were ranked based on gene number and the top 25 terms (Table S1) were further ranked based on fold enrichment. When terms were duplicated (e.g. “positive regulation of cell migration” and “cell migration”) (Table S1), only one was selected and presented in the top 10 list along with gene count, -log_10_ (*P*-value) and fold enrichment. The organ-specific term “nervous system development” was also not presented in the top 10 list.

### Statistical analyses

Parametric and statistical significance were determined using PRISM 9 (GraphPad Software, Inc., La Jolla, CA, USA). Parametric tests were used for datasets that passed the Shapiro–Wilk normality test. When sample size was too small for this test, non-normal distribution was assumed and non-parametric test was conducted. Two-tailed unpaired *t*-test, unpaired multiple Mann–Whitney *t*-tests or two-way analysis of variance (ANOVA) were performed as appropriate based on data distribution and as specified in the figure legends. When two-way ANOVA was performed, we utilized the multiple comparisons test (Tukey’s or Sidak’s) recommended in PRISM. A value of *P* < 0.05 was considered statistically significant. Graphs display mean and standard deviation, and each data point represents an independent mouse. Group sizes are indicated in figure legends.

## Availability of data and materials

Raw single-cell RNA sequencing data will be deposited in the NCBI Sequence Read Archive (SRA) and will be accessible from the NCBI Gene Expression Omnibus. Other raw data and processed single-cell RNA sequencing data are available at https://figshare.com/s/750f2493749963dfcb69.

### Supplementary information

Supplementary Information
