# Supplementary material for: Induction of osteoblast apoptosis stimulates macrophage efferocytosis and paradoxical bone formation
Source: Bone Res. 2024 Aug 5;12:43. doi: 10.1038/s41413-024-00341-9 (PMC11300627; doi:10.1038/s41413-024-00341-9)
Supplement: Supplementary file 1 — Supplementary Information [file 41413_2024_341_MOESM1_ESM.docx]

**Supplementary Information**

**Induction of osteoblast apoptosis stimulates macrophage efferocytosis and paradoxical bone formation**

Running title: Role of osteoblast death and clearance in bone

Lena Batoon^1^, Amy Jean Koh^1^, Susan Marie Millard^2^, Jobanpreet Grewal^1^, Fang Ming Choo^2^, Rahasudha Kannan^1^, Aysia Kinnaird^1^, Megan Avey^1^, Tatyana Teslya^1^, Allison Robyn Pettit^2^, Laurie Kay McCauley^1,3^*, Hernan Roca^1^*

^1^Department of Periodontics and Oral Medicine, University of Michigan, School of Dentistry, Ann Arbor, Michigan, USA, 48104

^2^Mater Research Institute, The University of Queensland, Brisbane, Queensland, Australia, 4102

^3^Department of Pathology, University of Michigan, Medical School, Ann Arbor, Michigan, USA, 48104


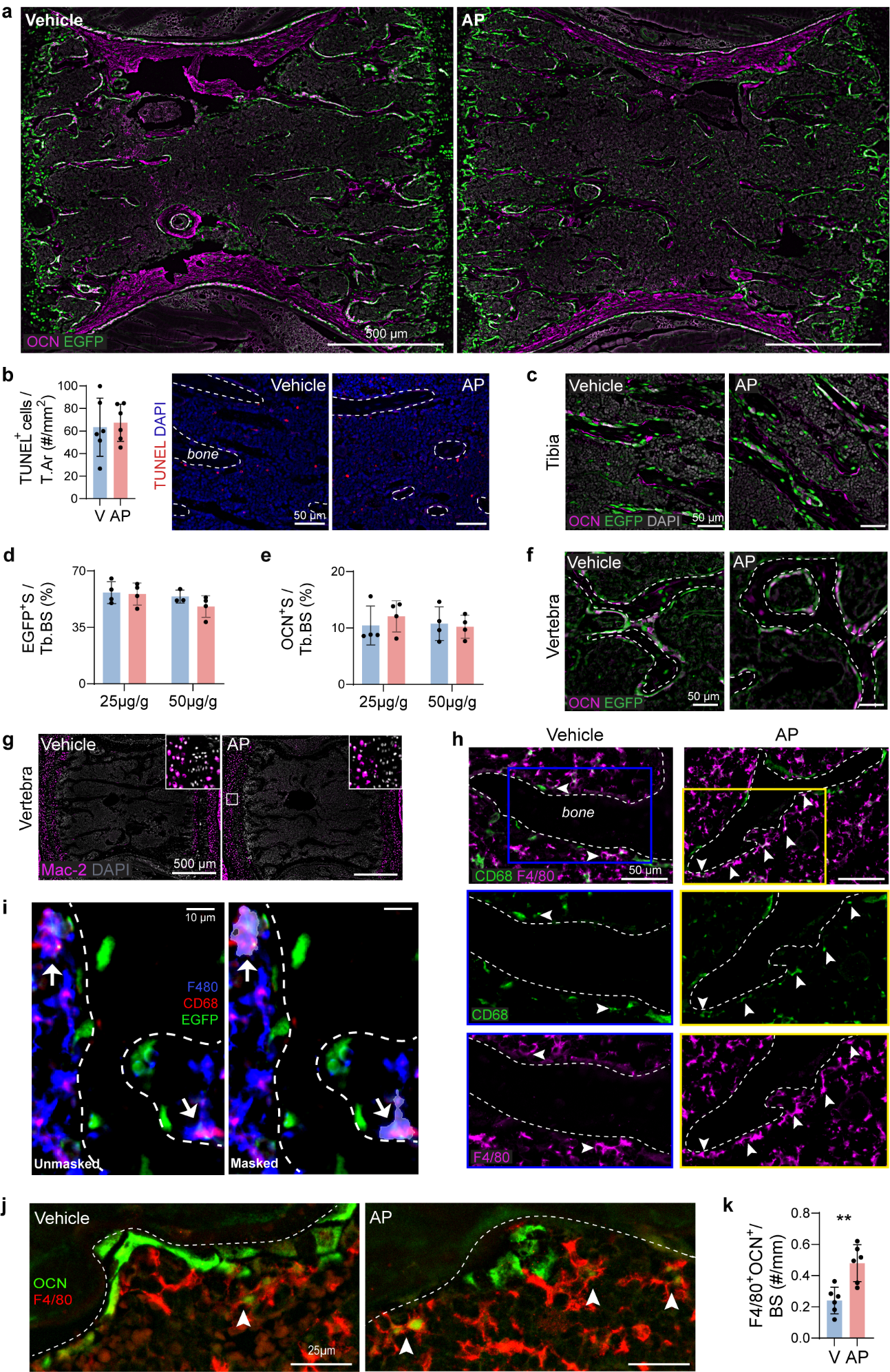


***Supplementary Fig 1. Other impacts of short-term AP treatment in OCNCre-iCasp9 mice.*** (**a**) Distribution of EGFP and OCN staining in the whole vertebra at 24h after short-term treatment with vehicle or AP. (**b**) Representative images taken in the metaphyseal region of the tibia showing staining and quantification of TUNEL^+^ cells. V = vehicle. Statistical significance was determined using two-tailed unpaired t-test. Error bars represent standard deviation. (**c**) Distribution of EGFP and OCN expression after short-term AP treatment in the tibia. Quantification of EGFP^+^ (**d**) and OCN^+^ (**e**) surface (S) per trabecular bone surface (Tb.BS) in the tibia of mice treated with one AP dose of 25µg/g or 50µg/g as well as their vehicle controls. (**f**) Representative images showing distribution of EGFP and OCN expression in the vertebrae of mice at 48hr following the last AP treatment. (**g**) Mac-2 expression in the vertebra showing pronounced expression in chondrocytes located in cartilaginous endplates. (**h**) Dual F4/80 and CD68 expression in the vertebrae showing each channel separately. Arrowheads indicate F4/80^+^CD68^+^ macrophages. (**i**) AIVIA software automated detection of F4/80^+^ macrophages (masked, white) containing both CD68 and EGFP signals (arrows). Only macrophages associated with bone were included in the analysis. (**j-k**) Representative images and quantification of F4/80^+^ cells containing OCN^+^ remnants (arrowheads) that are in close proximity to the bone surface at 24hr after three daily AP/vehicle injections. Statistical significance was determined using two-tailed unpaired t-tests. **p < 0.01. Error bars represent standard deviation. Each data point represents a single mouse. n= 6 mice/group.


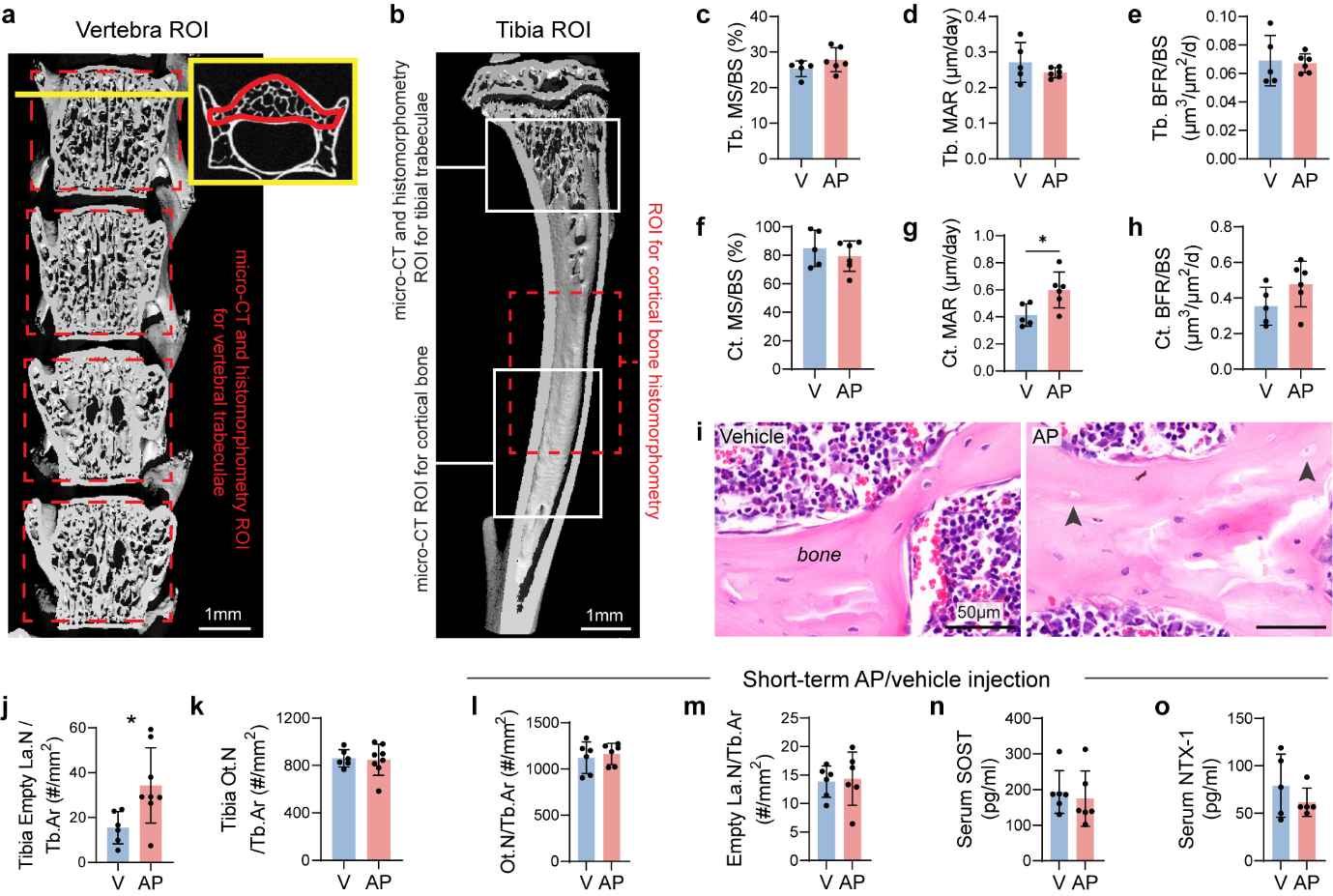


***Supplementary Fig 2. Regions of interest (ROI) assessed in bone tissues and other impacts of short- and long-term treatment.*** (**a**) Vertebral micro-CT analysis was performed in four lumbar vertebrae and all trabeculae within the vertebral body was examined. Histomorphometry in this tissue was also performed in all the trabeculae within the vertebral body. (**b**) Micro-CT analysis of the tibia was done in both the trabecular and cortical compartments. Both endocortical surfaces in the region indicated (red box) were included in tibial cortex dynamic histomorphometry. The cortical regions examined by micro-CT and histomorphometry slightly differ given the challenges with sectioning undecalcified plastic sections longitudinally. (**c-h**) Dynamic bone labeling analysis in the trabecular (Tb) or cortical (Ct) regions of the tibia showing mineralizing surface per bone surface (MS/BS), mineral apposition rate (MAR) and bone formation rate per bone surface (BFR/BS). V = vehicle. (**i**) H&E images of tibial trabeculae used to detect osteocytes and empty lacunae (arrowheads) following long-term AP/vehicle treatment. Enumeration of the number of (**j**) empty lacunae and (**k**) embedded osteocytes in tibial trabeculae. Enumeration of the number of (**l**) embedded osteocytes and (**m**) empty lacunae in vertebral trabeculae following short-term AP/vehicle treatment. Levels of circulating (**n**) SOST and (**o**) NTX-1 after short-term AP/vehicle treatment. Statistical significance was determined using two-tailed unpaired t-tests. *p < 0.05. Error bars represent standard deviation. Each data point represents a single mouse. (**c-h**) V n= 5 mice, AP n = 6 mice; (**i-k**) V n= 6 mice, AP n = 8 mice; (**l-n**) n = 6 mice/group; (**o**) n= 5 mice/group.

***
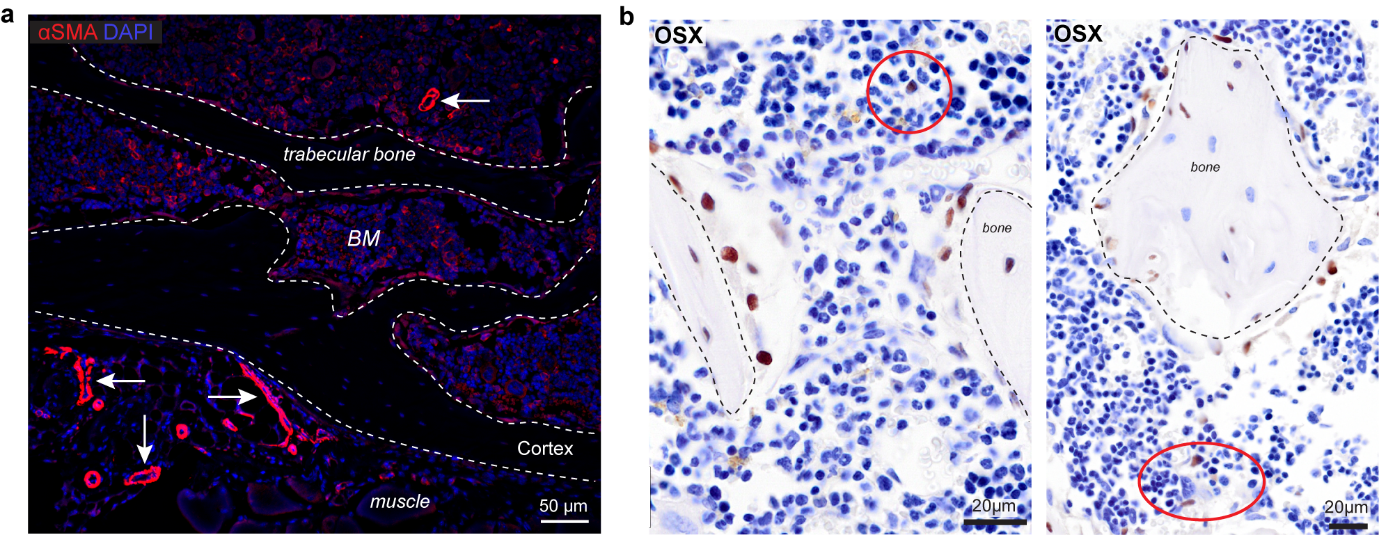
***

***Supplementary Fig 3. Distribution of αSMA and OSX expression in bone.*** (**a**) αSMA staining in the vertebra showing abundant expression in some vessels (arrows) and weaker but positive expression in some cells in the bone and marrow (BM). (**b**) Immunostaining for OSX expression showing that OSX^+^ cells were mainly located on the bone surface or embedded in the bone; however, some were also present in the bone marrow space (red circles). (**a**) n = 5 mice; (**b**) n = 3 mice.


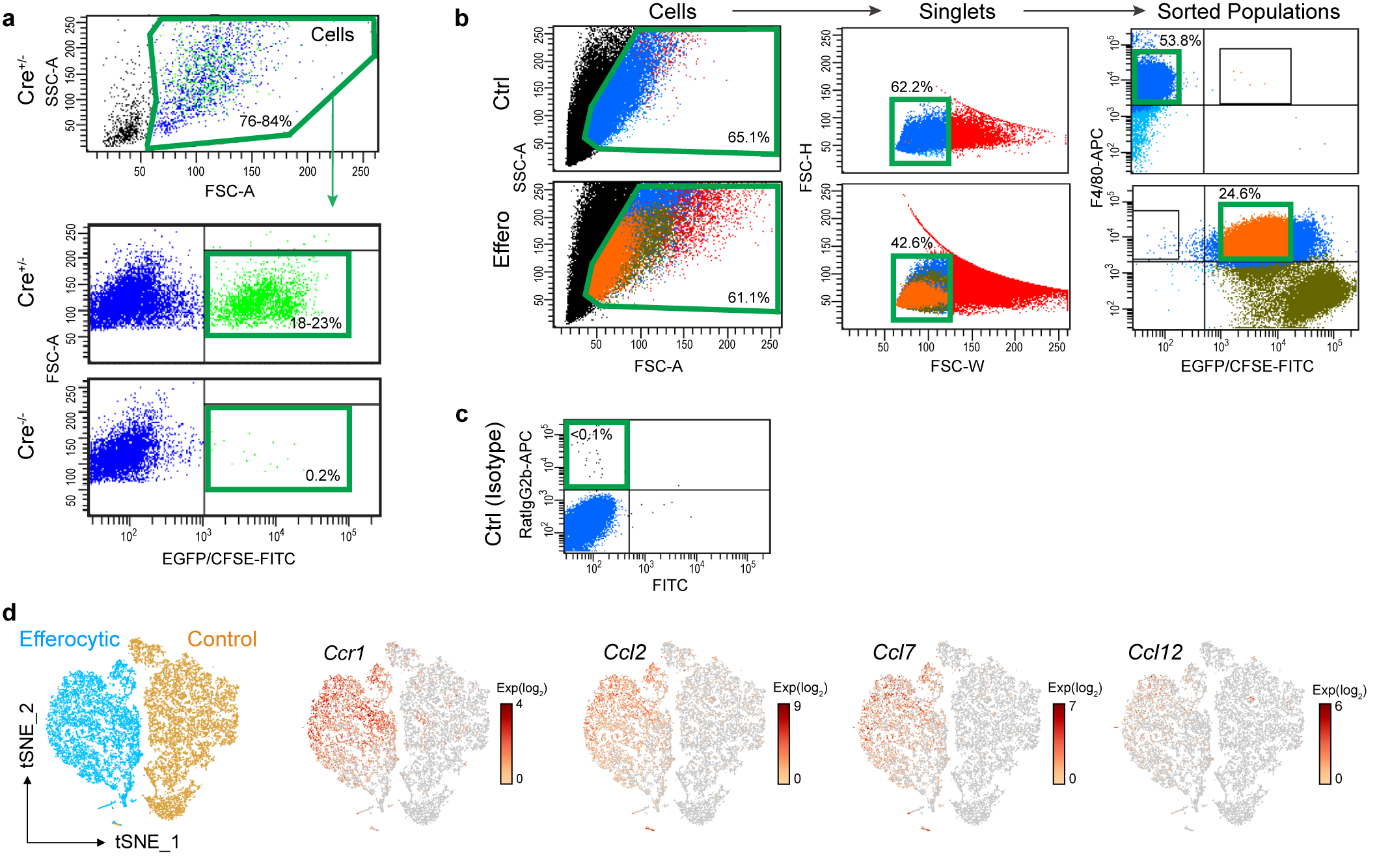


***Supplementary Fig 4. Single-cell RNA sequencing of in vitro generated efferocytic macrophages.*** (**a**) Gating strategy for EGFP^+^ calvarial osteoblast sorting. Debris were eliminated based on FSC-A/SSC-A and EGFP/CFSE^+^ cells were sorted. EGFP gating was established using cells from OSXCre^-/-^iCasp9^+/+^ calvaria that had undergone similar isolation procedure. Percentage of the populations per total events are presented. (**b**) Gating strategy for the sorting of control (Ctrl) and efferocytic (Effero) macrophages for single-cell RNA sequencing. A total of 128,922 “control” cells and 83,028 “efferocytic” cells were sorted. Percentage of the populations per total events are presented. (**c**) Isotype control for F4/80-APC used to establish the gates for sorting. (**d**) Dot plots showing the expression of *Ccr1*, *Ccl2*, *Ccl7* and *Ccl12* in “control” and “efferocytic” macrophage. n = 3-4 mice/group.


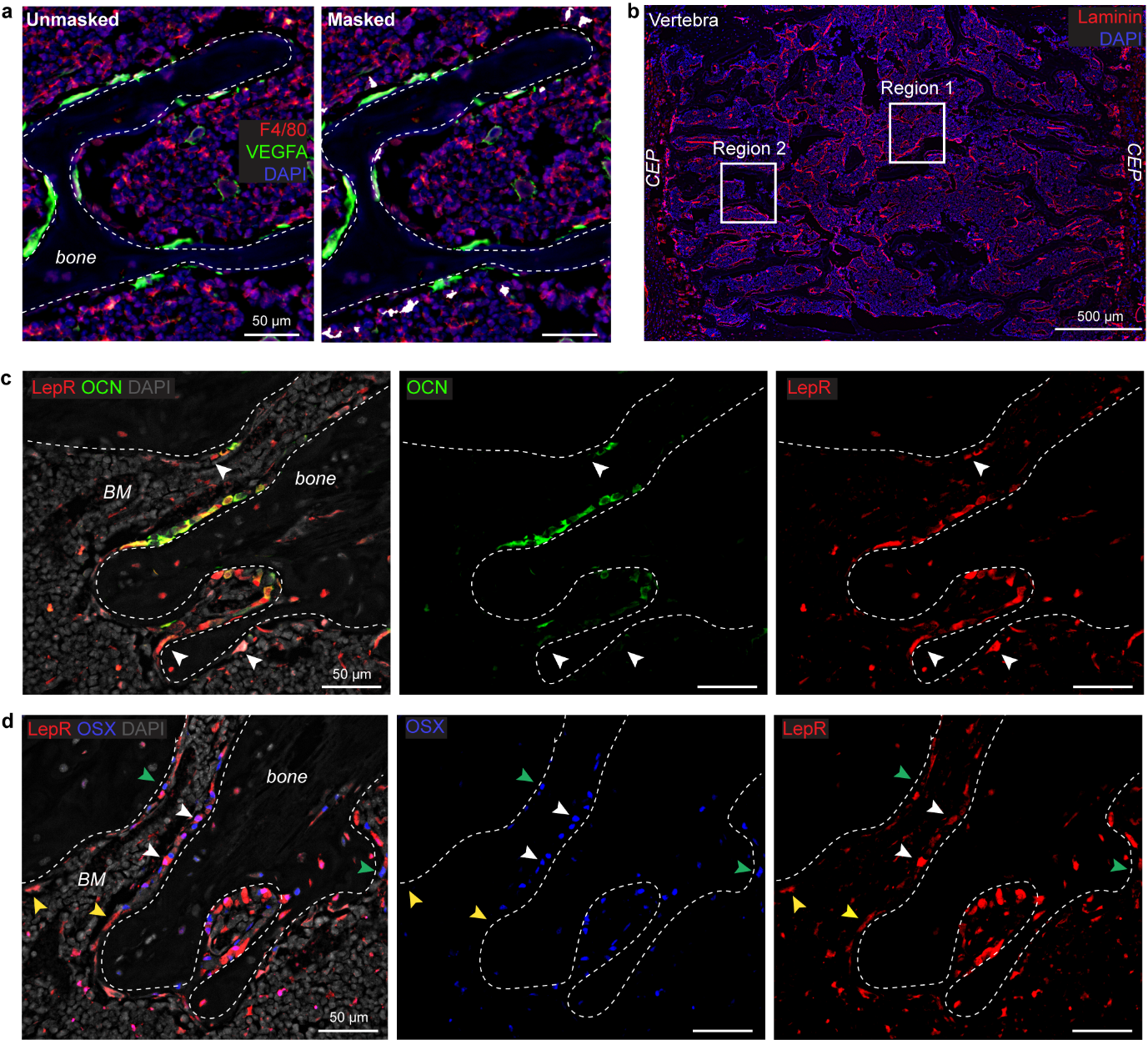


***Supplementary Fig 5. Other immunofluorescence staining detecting macrophages, vessels and osteolineage cells***. (**a**) AIVIA software automated detection of F4/80^+^VEGFA^+^ osteomacs (white) in the vertebra. Only double positive signals within one cell from the bone surface were included in the analysis. (**b**) Vertebral section stained with laminin and DAPI showing the regions where Fig. 6h representative images were taken from. CEP = cartilage endplate. (**c-d**) Sections from LepRiTom mice stained for (**c**) OCN or (**d**) OSX expression. (**c**) Arrowheads indicate LepR^+^ cells that are negative for OCN. (**d**) White arrowheads = LepR^+^OSX^+^ cells, yellow arrowheads = LepR^+^OSX^neg^, and green arrowheads = LepR^neg^OSX^+^. n = 5 mice.

**Supplementary Table 1. Top 25 GO Terms (Biological Process) ranked based on fold enrichment.**

| GO Term: Biological Process | Gene Count | P-value | Fold enrichment |
| --- | --- | --- | --- |
| angiogenesis | 91 | 1.20E-19 | 2.7 |
| positive regulation of cell migration | 78 | 3.30E-15 | 2.6 |
| negative regulation of cell proliferation | 115 | 8.30E-19 | 2.4 |
| cell migration* | 79 | 1.10E-11 | 2.2 |
| positive regulation of apoptotic process | 97 | 4.60E-13 | 2.1 |
| cell proliferation* | 74 | 2.00E-07 | 1.9 |
| cell adhesion | 123 | 1.50E-10 | 1.8 |
| apoptotic process* | 129 | 7.30E-10 | 1.7 |
| nervous system development* | 85 | 1.30E-06 | 1.7 |
| multicellular organism development | 194 | 1.10E-10 | 1.6 |
| phosphorylation | 112 | 7.30E-07 | 1.6 |
| intracellular signal transduction | 85 | 5.50E-06 | 1.6 |
| cell differentiation | 183 | 1.20E-07 | 1.5 |
| positive regulation of cell proliferation* | 108 | 2.20E-05 | 1.5 |
| protein phosphorylation | 107 | 6.20E-05 | 1.5 |
| positive regulation of gene expression | 103 | 3.10E-05 | 1.5 |
| positive regulation of transcription from RNA polymerase II promoter | 196 | 1.50E-06 | 1.4 |
| negative regulation of transcription from RNA polymerase II promoter | 167 | 1.10E-06 | 1.4 |
| lipid metabolic process | 118 | 4.20E-05 | 1.4 |
| negative regulation of transcription, DNA-templated | 106 | 4.70E-04 | 1.4 |
| negative regulation of apoptotic process | 103 | 1.00E-03 | 1.4 |
| positive regulation of transcription, DNA-templated | 110 | 1.70E-03 | 1.3 |
| protein transport | 95 | 5.70E-03 | 1.3 |
| cell cycle | 88 | 5.60E-02 | 1.2 |
| signal transduction | 178 | 4.50E-02 | 1.1 |

**Terms that were not presented in the top 10 list due to being similar/closely-related with another term or for being a process specific to a different organ.*

**Supplementary Table 2. Immunofluorescence and immunohistochemistry antibodies.**

| Antibody | Source | Identifier | Dilution | Incubation time | Antigen retrieval |
| --- | --- | --- | --- | --- | --- |
| Amphiregulin | Thermo Fisher | PA5-102501 | 1:250 | 2h | Sodium Citrate (pH 6) |
| CD68 | Abcam | ab125212 | 1:250 | 2h | Proteinase K (50µg/ml) |
| Col1a1 | USBiological | C7510-13 | 1:300 | 2h | Trypsin (0.1%) |
| Donkey anti-goat- AlexaFluor488 | Thermo Fisher | A11055 | 1:200 | 1h | Not applicable |
| EGFP/GFP | Abcam | ab13970 | 1:500 | 2h | Proteinase K (50µg/ml) |
| F4/80 [CI:A3-1] | Abcam | ab6640 | 1:200 | 1.5h | Proteinase K (50µg/ml) |
| Goat anti-chicken-AlexaFluor488 | Abcam | ab150169 | 1:200 | 1h | Not applicable |
| Goat anti-rabbit IgG (H+L) Antibody-biotin | Vector Laboratories | BA-1000-1.5 | 1:600 | 1h | Not applicable |
| Goat anti-rabbit-AlexaFluor680 | Thermo Fisher | A21076 | 1:200 | 1h | Not applicable |
| Goat anti-rat-AlexaFluor647 | Biolegend | 405416 | 1:200 | 1h | Not applicable |
| Goat anti-rat-AlexaFluorCy3 | Thermo Fisher | A10522 | 1:200 | 1h | Not applicable |
| Ki67 | Abcam | ab15580 | 1:500 | 2h | Trypsin (0.1%) |
| Laminin | Novus | NB300-144B | 1:400 | 2h | Pepsin (0.5%) |
| Ly6G [EPR22909-135] | Abcam | ab238132 | 1:1000 | Overnight | Proteinase K (50µg/ml) |
| Mac-2 [eBioM3/38 (M3/38)] | eBioscience | 14-5301 | 1:600 | 2h | None |
| Osteocalcin | Thermo Fisher | PA578870 | 1:200 | 2h | Proteinase K (50µg/ml) |
| Osterix [EPR21034] | Abcam | ab209484 | 1:1000 | 2h | None |
| Sclerostin | R&D Systems | AF1589 | 1:100 | 2h | Trypsin (0.1%) |
| Streptavidin (HRP) | Abcam | ab7403 | 1:5000 | 1h | Not applicable |
| αSMA | Abcam | ab5694 | 1:500 | Overnight | Sodium Citrate (pH 6) |
